# Simulation of the impact of rifampicin on darunavir/ritonavir PK and dose adjustment strategies in HIV-infected patients: a population PK approach

**DOI:** 10.7448/IAS.17.4.19586

**Published:** 2014-11-02

**Authors:** Laura Dickinson, Alan Winston, Marta Boffito, Saye Khoo, David Back, Marco Siccardi

**Affiliations:** 1 Department of Molecular & Clinical Pharmacology, University of Liverpool, Liverpool, UK; 2 Faculty of Medicine, Imperial College, London, UK; 3 Department of HIV & Genitourinary Medicine, Imperial College Healthcare NHS Trust St Mary's Hospital, London, UK; 4 St Stephen's Centre, Chelsea & Westminster Foundation Trust, London, UK

## Abstract

**Introduction:**

Treatment of HIV/TB co-infection is challenging due to high drug–drug interaction potential between antiretrovirals and rifamycins, such as rifampicin (RIF). The PK interaction between darunavir/ritonavir (DRV/RTV) and RIF has not been studied. Utilizing other protease inhibitor data, population PK modelling and simulation was applied to assess the impact of RIF on DRV/RTV PK and generate alternative dosing strategies to aid future clinical trial design.

**Materials and Methods:**

A previously developed model describing DRV/RTV PK including data from three studies in HIV patients was used [n=51, 7 female, DRV/RTV 800/100 mg (n=32) or 900/100 mg once daily (*qd*; n=19) [1]. The PK interaction between DRV/RTV and RIF was assumed to mimic that observed in HIV-infected, TB negative patients receiving lopinavir (LPV)/RTV (n=21) [2]. Simulations of DRV/RTV 800/100 mg *qd* (n=1000) were performed (-RIF). The model was adapted to increase the typical value of apparent oral clearance (CL/F) by 71% and 36% and decrease relative bioavailability (F) by 20% and 45% for DRV and RTV, respectively [2]; 1000 simulations were generated (+RIF). Dose adjustments of DRV/RTV 1200/200 mg *qd*, 800/100 mg and 1200/150 mg twice daily (*bid*) were simulated to overcome the interaction. DRV trough (C_trough_) for each dosing scenario was compared to the reference (-RIF) by GMR (90% CI).

**Results:**

DRV and RTV were described by a 1 and 2-compartment model, respectively. A maximum effect model, with RTV inhibiting DRV CL/F, best described the relationship between the drugs. Compared to the reference (-RIF), simulated DRV C_trough_ was 70%, 46% and 20% lower for 800/100 mg *qd*, 1200/200 mg *qd* and 800/100 mg *bid* all +RIF, respectively. C_trough_ was 38% higher with 1200/150 mg *bid* +RIF (Table 1).

**Conclusions:**

Modelling and simulation was used to investigate the theoretical impact of RIF on DRV/RTV PK. Based on simulations, 800/100 mg and 1200/150 mg both *bid* could largely overcome the impact of the interaction. However, the risk of increased RTV-related side effects and higher pill burden should be considered. *In vitro* work is ongoing to develop a physiologically based model characterizing the interaction and informing simulations.

**Table 1:** Summary of model simulated DRV C_trough_ concentrations in the absence and presence of RIF and following dose adjustment in combination with RIF. The changes in simulated DRV C_trough_ are presented as GMR (90% CI)

Regimen	Geometric mean (90% CI)	GMR (90% CI)
800/100 mg *qd* -RIF (reference)	1.642 (1582–1.702)	–
800/100 mg *qd* +RIF	0.486 (0.461–0.511)	0.296 (0.293–0.299)
1200/200 mg *qd* +RIF	0.883 (0.839–0.927)	0.538 (0.533–0.542)
800/100 mg *bid* +RIF	1.311 (1.262–1.359)	0.798 (0.761–0.837)
1200/150 mg *bid* +RIF	2.270 (2.191–2.349)	1.383 (1.319–1.449)
